# Metastatic Urothelial Carcinoma from Transplanted Kidney with Complete Response to an Immune Checkpoint Inhibitor

**DOI:** 10.1155/2020/8881841

**Published:** 2020-12-23

**Authors:** Ryan S. Chiang, Ashton A. Connor, Brant A. Inman, Wen-Chi Foo, David N. Howell, John F. Madden, Matthew J. Ellis, Aparna S. Rege, Michael R. Harrison

**Affiliations:** ^1^Department of Medicine, Stanford University Medical Center, Stanford, CA, USA; ^2^Division of Abdominal Transplant Surgery, Department of Surgery, Duke University School of Medicine, Durham, NC, USA; ^3^Division of Urology, Department of Surgery, Duke University School of Medicine, Durham, NC, USA; ^4^Department of Pathology, Duke University School of Medicine, Durham, NC, USA; ^5^Divisions of Nephrology and Abdominal Transplant, Departments of Medicine and Surgery, Duke University School of Medicine, Durham, NC, USA; ^6^Division of Medical Oncology, Department of Medicine, Duke University School of Medicine, Durham, NC, USA

## Abstract

**Background:**

Donor-derived malignancy is a rare complication in patients who undergo organ transplant. Approaches to treatment have largely been individualized based on clinical circumstances given the lack of evidence-based guidelines, with therapeutic options ranging from discontinuation of immunosuppression and transplantectomy to the addition of chemotherapy or radiotherapy. *Case Presentation*. Herein, we describe a 60-year-old woman with metastatic donor-derived upper tract urothelial carcinoma (UTUC) discovered nine years postrenal transplant. Molecular diagnostic studies using polymerase chain reaction amplification of short tandem repeat alleles and HLA tissue typing proved that the urothelial carcinoma originated from donor tissue. She achieved sustained complete remission with transplant nephroureterectomy, retroperitoneal lymphadenectomy, immunosuppression withdrawal, and immunotherapy with pembrolizumab. Routine radiologic surveillance has demonstrated 15-month progression-free survival to date off pembrolizumab, and she is now under consideration for retransplantation.

**Conclusions:**

Immunotherapy using checkpoint inhibitors can serve as a novel treatment option for patients in the clinical predicament of having a solid organ transplant and simultaneous metastatic malignancy. In this report, we also discuss the oncogenic potential of BK virus, the use of checkpoint inhibitors in urothelial carcinoma, and the feasibility of retransplant for this patient population.

## 1. Background

Nearly 36,500 transplants were performed in the U.S. in 2018, of which renal transplants comprised the majority at over 21,100 [1]. Solid organ transplant recipients are at higher risk of malignancies, due partly to immunosuppressive medications, decreased immune system surveillance, and risk of oncogenic viral infection [2, 3]. Donor-related malignancy is relatively rare and can be categorized as donor-transmitted if presenting shortly after transplant or donor-derived if many years later [4–6]. There are several reported cases of donor-related malignancies achieving complete remission after transplant organ resection, immunosuppression withdrawal, and occasionally chemotherapy or radiotherapy [7–10]. Features reported in 36 published allograft upper tract urothelial carcinoma (UTUC) cases include long latency (mean 10 years) from transplant to disease presentation, immunosuppression and BK infection as risk factors, and highly aggressive tumor biology [10]. Ortega et al. described a case of donor-derived UTUC discovered nine years postrenal transplant and complicated by BK viremia six years posttransplant successfully managed with transplant nephrectomy and immunosuppression withdrawal [11].

Here, we describe a patient who presented with high-grade stage IV UTUC of donor origin ten years after renal allotransplantation. Our patient was treated with transplant nephroureterectomy, retroperitoneal lymphadenectomy, immunosuppression withdrawal, and immunotherapy with pembrolizumab, achieving a sustained complete response.

## 2. Case Presentation

A 51-year-old Chinese woman with dialysis-dependent end-stage renal disease secondary to IgA nephropathy underwent deceased donor kidney transplant. Donor medical history did not include malignancies. Recipient medical history included vulvar and cervical intraepithelial dysplasia. She had stable creatinine 2.0 mg/dL on tacrolimus, mycophenolate, and prednisone, despite chronic BK viral infection.

**Figure 1:**
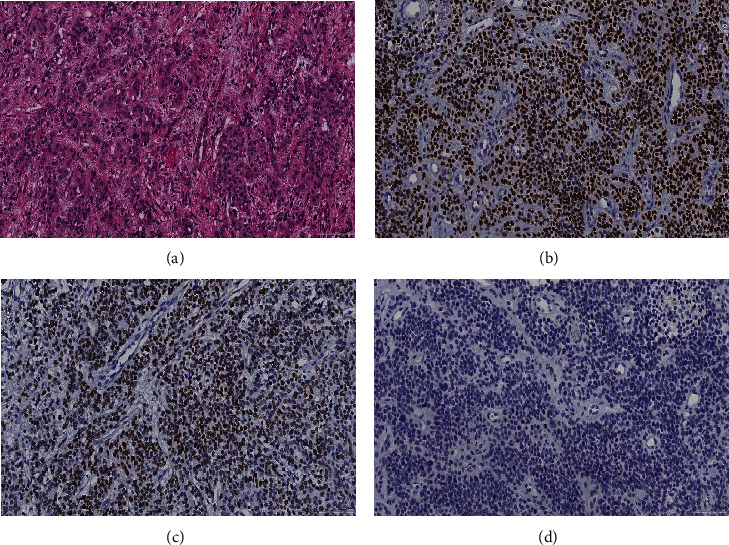
(a) The nephrectomy specimen demonstrated infiltrative nests of tumor cells with prominent nucleoli, moderate pleomorphism, and scattered mitoses (H&E). (b) Nuclear expression for p63 in the tumor cells supports the diagnosis of urothelial carcinoma. (c) SV40/BKV stain showing positive staining in the tumor cells. (d) PAX8 is negative in tumor cells, excluding renal cell carcinoma.

Nine years posttransplant, she presented with a several-month history of episodic severe lower abdominal pain. Abdominal computed tomography (CT) revealed a 5.8 × 5.5 × 4.6 cm renal allograft hilar mass, with FDG-avid supraclavicular, mesenteric, retroperitoneal, pulmonary, and hepatic lesions on PET. This imaging, urine cytology, allograft biopsy, and diagnostic laparoscopy were concordant for stage IV UTUC. She underwent transplant nephrectomy and retroperitoneal lymphadenectomy, with final pathology demonstrating pT4N2M0R1 UTUC, with malignant epithelium, high-grade atypia, interstitial fibrosis, tubular atrophy, and extranodal extension, with the largest foci measuring 1.5 cm. There was neither rejection nor IgA nephropathy recurrence. Immunohistochemistry (IHC) stains were negative for C4d and PAX8 and positive for p63, GATA3, CK20, p16, and SV40/BKV (see Figure 1), consistent with UTUC with BK viral protein expression. Next-generation sequencing results were notable for microsatellite-stable, undeterminable tumor mutational burden, EGFR amplification, KDM6A L945fs∗25, and RAF1 amplification. PD-L1 IHC analysis showed a combined positive score of zero (therefore, PD-L1-negative). Chimerism was assessed by PCR amplification of fifteen short tandem repeats and by HLA tissue typing, both showing concordance between donor and malignancy, but not recipient, implying allograft origin.

**Figure 2:**
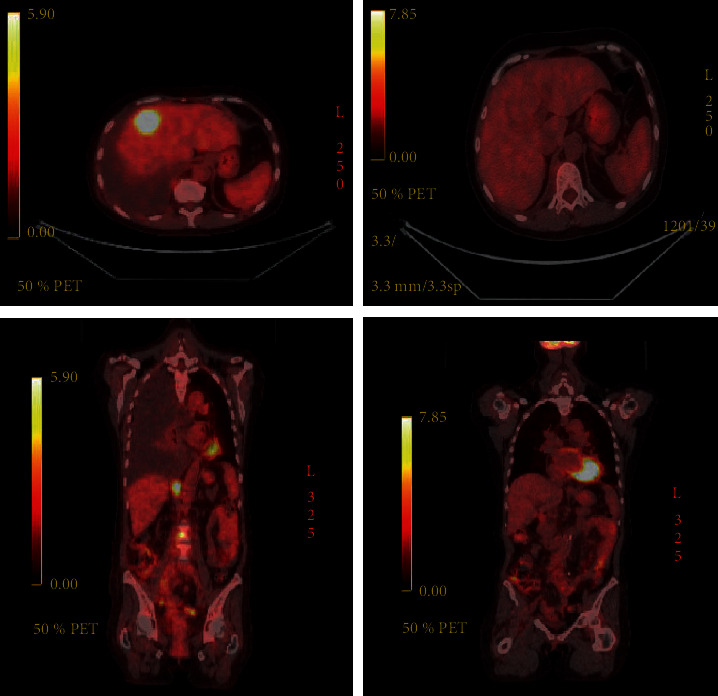
PET/CT scans showing improvement in metastatic disease with reduction in hepatic mass and decreased retroperitoneal, pelvic, and iliac lymphadenopathy after 4 cycles of pembrolizumab.

Despite stopping immunosuppression, disease progression was seen on restaging CT scans two months later. Multidisciplinary cancer conference consensus was for PD-1 blockade with pembrolizumab. She received 28 cycles of pembrolizumab 200 mg IV every three weeks over about 19 months, achieving complete radiological response, with 15-month progression-free survival to date off therapy (see Figure 2). Surveillance imaging continues at 3-month intervals.

## 3. Discussion

We present a case of post-renal-allotransplantation donor-derived, disseminated UTUC that achieved complete response with transplant nephrectomy, retroperitoneal lymphadenectomy, immunosuppression cessation, and immunotherapy. There have been several cases described in the literature where withdrawal of immunosuppression has been sufficient for treatment of squamous cell carcinomas, undifferentiated epithelioid tumor, and donor-derived urothelial carcinoma in renal transplant patients [11–14]. To our knowledge, this is the first reported case where a patient who developed donor-derived urothelial carcinoma achieved complete response on immune checkpoint inhibitor therapy and is now considering a repeat renal transplant. This case adds to the literature on a rare complication of kidney transplantation, with important features being BK viral expression in cancer cells, the indications for immunotherapy, and the consideration for redo transplant.

Many viruses have been implicated to promote oncogenesis through a variety of mechanisms not limited to tumor suppressor protein degradation, direct genome integration, and cell cycle dysregulation [15–18]. BK polyomavirus is a widespread double-stranded DNA virus that tends to pathologically manifest in immunocompromised individuals such as those who undergo organ transplantation through viral reactivation [19]. In recent years, BK polyomavirus has emerged as a source of malignant transformation in humans with improved understanding of its carcinogenic pathophysiology. The primary oncogenic driver seems to be persistent latent infection via genome integration which leads to overexpression of the viral oncoprotein and large T antigen and subsequently promotes unwarranted cellular proliferation [20–23]. Several case reports have highlighted the potential for BK viremia to predispose patients to posttransplant urothelial carcinoma through persistent infection coupled with chronic immunosuppression which serves as an ideal breeding ground for cancer development [24, 25]. A fully intact immune system is often sufficient for resuppression of BK viremia and may explain why some patients with donor-derived malignancy experience complete cancer remission upon immunosuppression discontinuation and transplantectomy alone. PD-1 plays a significant role in T cell exhaustion during chronic viral infections which can serve as one potential mechanism of efficacy for pembrolizumab as the blockade can lead to restoration of T cell function. However, the exact duration of BK viremia could not be established for our patient since she had received only one BK virus quantitative PCR which showed detectable levels several months prior to the official diagnosis of metastatic UTUC. Repeat measurement at the time of cancer diagnosis demonstrated significant elevation in the number of viral copies. PD-L1 testing performed after diagnosis was negative which makes the mechanism of favorable efficacy for pembrolizumab unclear in this case.

Standard therapy for metastatic urothelial carcinoma has historically been combination cisplatin-based chemotherapy in those who are cisplatin-eligible [26]. For those with platinum-refractory urothelial carcinoma, pembrolizumab has been an effective option with better overall survival rates as well as lower rates of adverse events when compared to standard chemotherapy regimens [27]. Pembrolizumab is an immune checkpoint inhibitor which functions by blocking the PD-1 receptor to allow a greater natural immune response against tumors [28]. Based on the findings of KEYNOTE-52, a multicenter, single-arm, phase 2 study, pembrolizumab can also serve as an effective and safe first-line therapy option in patients who are cisplatin-ineligible [29]. Many clinical trials have excluded patients with solid organ transplants who develop metastatic cancers from the use of checkpoint inhibitors due to concerns for graft rejection [30, 31]. One systematic review of 57 patients found that the use of immune checkpoint inhibitors had no significant association with graft rejection and can have clinically relevant response rates for treating metastatic cancer [30]. A recently published case series found that although immune checkpoint inhibitors can serve as a viable therapeutic option for transplant patients, prospective studies are warranted given the potential risk of allograft rejection [32]. There is currently limited data on the safety and efficacy of immune checkpoint inhibitor rechallenge after withdrawal due to complete response, but restarting immune checkpoint inhibitor therapy after progression often results in very low response rates [33, 34]. Given these studies, we could potentially provide her with another renal transplant while reinitiating pembrolizumab followed by close monitoring for both signs of graft rejection as well as signs of cancer recurrence.

There is little published experience on redo renal allotransplantation following complete remission of donor-derived malignancies. Occult metastatic disease may resurface with reinstitution of immunosuppression. Yet, most patients can safely undergo solid organ transplantation after two years of disease-free survival following treatment of most malignancies, including renal cell carcinoma. The indications for redo kidney transplant include improved quality and quantity of life compared with dialysis by eliminating scheduled dialysis sessions and minimizing cardiovascular complications [35–37]. Georgieva et al. describe 3 patients with donor-related malignancies who successfully underwent retransplantation after achieving complete remission, implying that it may be safe [7].

## 4. Conclusion

As the need for solid organ transplantation continues to increase, the prevalence of donor-related malignancies can be expected to also rise. More evidence is required to inform management of these uncommon malignancies that currently have high morbidity and mortality. This case includes discussion of novel etiology, investigation, treatment, surveillance, and postremission redo transplantation. With increased published experience, we may advance beyond individualized approaches to having evidence-based guidelines.

## Data Availability

No data was used to support this study.
